# Editorial: Rising stars in plant metabolism and chemodiversity 2022 - phenylpropanoid metabolism and regulation

**DOI:** 10.3389/fpls.2023.1159100

**Published:** 2023-02-21

**Authors:** Yunjun Zhao, Chang Liu, Chien-Yuan Lin, Quanzi Li

**Affiliations:** ^1^ CAS Center for Excellence in Molecular Plant Sciences, Shanghai Institute of Plant Physiology and Ecology, Chinese Academy of Sciences (CAS), Shanghai, China; ^2^ State Key Laboratory of Tree Genetics and Breeding, Northeast Forestry University, Harbin, China; ^3^ The Center for Basic Forestry Research, College of Forestry, Northeast Forestry University, Harbin, China; ^4^ Environmental Genomics and Systems Biology Division, Lawrence Berkeley National Laboratory (LBNL), Berkeley, CA, United States; ^5^ State Key Laboratory of Tree Genetics and Breeding, Chinese Academy of Forestry, Beijing, China

**Keywords:** phenylpropanoid metabolism, epigenetic regulation, lignin, flavonoid, anthocyanin, proanthocyanidin

Phenylpropanoids are a diverse group of specialized metabolites that contribute to the basic process of plant growth and development, as well as to the plant–environment interactions. They are biosynthesized from the shikimic acid pathway *via* the aromatic amino acid phenylalanine or tyrosine in certain plants (Deng and Lu, 2017). These amino acids provide phenylpropanoids a C6-C3 (a phenyl group linked to a 3‐C propane side chain) skeleton, producing derivatives with one, two or more aromatic rings, each ring with a variable substitution pattern and with different modifications of the C3 side chain.

The phenylpropanoid metabolism yields a huge quantity of compounds with multiple biological activities, such as flavonoids (e.g. flavanones, flavones, flavonols, flavanols, anthocyanidins, and isoflavones), which participate in organ pigmentation, UV protection and plant-microbe interactions; lignin, which is involved in the mechanical support and waterproofing of plant cell walls; condensed tannins, which give the fruit and its products important organoleptic properties, like astringency, bitterness, and colour stability; and phytoalexins, which are active in resisting herbivores and infectious pathogens (Deng and Lu, 2017). Along with their biological functions, phenylpropanoids are economically significant metabolites. They are of interest for their numerous pharmacological and industrial applications, for example, several of which are considered high-value biochemicals used in the production of perfumes, pharmaceuticals, and biopolymers (Lin and Eudes, 2020). Moreover, the phenylpropanoid-based polymers such as lignin and suberin are considered potential targets for creating recalcitrant forms of carbon in plants, towards carbon sequestration in soil and biomass (Eckardt et al., 2023). Hence, phenylpropanoids research is beneficial to provide a promising plant-based solution for carbon neutrality.

Over the past decades, elaborate molecular mechanisms for regulating the phenylpropanoid pathway at multiple levels have been extensively studied. Phenylpropanoid metabolism has been revealed to be modulated by multiple regulatory mechanisms, including transcriptional, post‐transcriptional, post‐translational, and epigenetic regulation, and through a variety of signaling pathways, such as phytohormone, biotic stress, and abiotic stress signaling pathways (Dong and Lin, 2021). This Research Topic sought to collect recent findings in all aspects of phenylpropanoid metabolism and regulation.

Phenylpropanoids exhibit extraordinary complexity and high‐level plasticity in different species, developmental stages and in response to environmental stimuli. Significantly, many of phenylpropanoids can be specific to only one or a few plant species; it’s, therefore, necessary to have comprehensive analyses of phenylpropanoids among various species to expand our knowledge gained mainly from model plant species. Wang et al. (in this Research Topic) identified and quantified 133 flavonoids within the seed coat of four different testa-colored peanut cultivars and proposed several MYB-like transcription factors (TFs), an anthocyanidin reductase (ANR), and a UDP-glycosyltransferase (AhUGT236) were implicated in the testa pigmentation based on RNA-seq and gene co-expression network analysis.

A group of TFs including MYBs, NACs, MBW ternary complex composed of three classes of regulators, namely R2R3‐MYB TFs, basic helix‐loop‐helix (bHLH) TFs, and WD40 Repeat (WDR) proteins, and other TFs were believed to be critical in regulating the structural genes of lignin or flavonoid biosynthesis. Cheng et al. (in this Research Topic) have characterized a novel bHLH TF, VvibHLH93, as a negative regulator in the proanthocyanidins (PAs) biosynthesis route of grapevine, and found that VvibHLH93 could target a wide range of structural genes and TFs genes in flavonoid pathway. The findings of this study essentially complement existing knowledge on the regulation of PAs. Lu et al. (in this Research Topic) have found a cluster of R2R3-MYB TF, XsMYB113s, and showed they likely control the progressive color changes during yellowhorn flower developments through positively regulating the anthocyanin biosynthesis genes.

In addition to transcriptional regulation, there is growing interest in the role of epigenetic regulation in controlling phenylpropanoid metabolism. Epigenetic regulations, such asDNA methylation and demethylation, have been implicated in anthocyanin biosynthesis and associated with pigmentation. Lu et al. (in this Research Topic) showed the methylation status of CHH on the transposon element near the *XsMYB113-1* influenced its expression and dynamic epigenetic regulation of the *XsMYB113-1* affected anthocyanins buildup along with color changes during yellowhorn flower development.

Environment stimuli frequently trigger phenylpropanoids biosynthesis. Therefore, lignin biosynthesis can be affected by environmental variations due to climate change. Chen et al. (in this Research Topic) studied the relationship between lignin biosynthesis and 19 environmental factors in natural birch. They discovered that the lignin content in birch wood was negatively correlated with climate temperature. They also showed that DNA methylation levels in the promoter regions of two key NAC TFs, *BpNST1/2* and *BpSND1*, were variable in birch trees grown in different environments, and suggest that DNA hypermethylation may repress the expression of these genes and thus negatively regulate lignin biosynthesis. This study provides evidence that environmental signals can lead to epigenetic variations that cause changes in lignin biosynthesis.

What these four research articles have in common is that their research objects are all non-model plants of significant economic and ecological value. As more plant species for which full-genome sequences are becoming available, it will benefit the studies to explore the functions of phenylpropanoid metabolites in more plants and enrich the diversification of phenylpropanoid research on a multi-species level.

Phenylpropanoid metabolism is often connected to a complex interaction between cell signaling and environmental influences. The studies in the current Research Topic have greatly advanced our understanding of phenylpropanoid metabolic regulation in plants *via* the exploration of metabolic diversity, transcriptional regulation, epigenetic modification, and environmental interaction. In the future, the ability to bridge the fields of molecular biology, epigenetics, chemistry, evolutionary biology, and molecular ecology to resolve the complexity of phenylpropanoid metabolism cooperatively will provide high-resolution information on its metabolic regulatory networks.

## Author contributions

All authors listed have made a substantial, direct, and intellectual contribution to the work and approved it for publication.
